# Blood metabolic profile and complete blood count of sheep during late pregnancy and lactation

**DOI:** 10.5194/aab-69-337-2026

**Published:** 2026-06-22

**Authors:** Zvonko Antunović, Josipa Kučan, Željka Klir Šalavardić, Mislav Djidara, Josip Novoselec

**Affiliations:** 1 Department of Animal Production and Biotechnology, Faculty of Agrobiotechnical Sceinces Osijek, University of J. J. Strossmayer in Osijek, V. Preloga 1, 31000 Osijek, Croatia

## Abstract

The research was performed on 30 adult clinically healthy Lika Pramenka sheep, aiming to determine the influence of late pregnancy and lactation on blood metabolic profile and complete blood count. Sheep were, on average, 5 years old, in the fourth lactation. The sheep were fed meadow hay at an amount of 1.8 $\mathrm{kg}\;d^{-1}$ and oat at an amount of 800 $g\;d^{-1}$ . Blood was sampled during the late-pregnancy stage of the sheep (on the 20th day before lambing) and during the lactation period on the 40th, 70th and 100th days. Complete blood counts determined in this research were the blood cell counts; hemoglobin (HGB), hematocrit (HCT) and erythrocyte constants; and differential blood count. Biochemical analyses in sheep blood serum determined concentrations of minerals, metabolites and enzyme activities. This research proved that the late pregnancy and lactation had a significant influence on the indicators of the sheep's blood metabolic profile and complete blood count. Significant changes in complete blood count were particularly expressed in late-pregnancy and lactating sheep in the first measurements, especially in terms of values of RBCs (red blood cells), HGB, HCT, PLTs (platelets), lymphocytes and neutrophils; indicators of energy (NEFAs or non-esterified fatty acids, glucose and triglycerides), protein (TP or total protein, ALB or albumin, urea) and mineral status (Ca, P-inorganic); and liver status (changes in most liver enzymes). Moreover, the quality of feed (protein and mineral content) plays an important role in the determination of the blood metabolic profile and complete blood count of sheep during late pregnancy and lactation. Therefore, this research suggests that studies should include late pregnancy and lactation and feed quality in the determination of the metabolic profile and complete blood count of sheep's blood in order to obtain the best possible information on the herd.

## 1 Introduction

Determination of sheep's blood indicators, primarily complete blood count and biochemical, mineral and enzymatic status, is important with regard to the quality monitoring of physiological and pathological processes in sheep (Očenaš et al., 2025). The mentioned indicators refer to the blood metabolic profile of sheep (Van Saun, 2023). Analysis of complete blood count determines the blood cell counts (erythrocytes, leukocytes, and platelets), hemoglobin, hematocrit, erythrocyte constants (mean corpuscular volume (MCV), mean corpuscular hemoglobin (MCH) and mean corpuscular hemoglobin concentration in the erythrocytes (MCHC)) and differential blood count, which are essential for early diagnosis and prognosis and the monitoring of therapeutic progression in livestock (Korelidou et al., 2026; Yang et al., 2024; Antunović et al., 2017; Polizopoulous, 2010). Biochemical status of blood is determined through energy, protein and mineral profiles, while the liver status is determined by the enzyme activity (AST or aspartate aminotransferase, ALT or alanine aminotransferase, GGT or γ -glutamyl transferase) (Ljubičić et al., 2022). Energy status includes determination of non-esterified fatty acid (NEFA), glucose, cholesterols and triglycerides, while protein status can be monitored by determining blood concentrations of total protein (TP), albumin, globulins and urea (Herdt, 2000; Van Saun, 2023). Blood metabolic profile (BMP) and complete blood count are used in evaluation of the clinical status, nutritional status, deficit condition, treatment monitoring and prognostics, and it is useful when metabolic disorders in sheep should be diagnosed (Beigh et al., 2018; Antunović et al., 2004). It is affected by numerous factors, primarily by nutrition, physiological status and environmental conditions, and, when interpreted as a whole, it serves for quantitative and qualitative monitoring of animal productivity, elimination of reproductive disorders and prevention of numerous subclinical diseases (Akkaya et al., 2020; Van Saun, 2023). Physiological stages influence the blood metabolic profile and complete blood count indicators since pregnancy, especially the last phase of pregnancy, and lactation are the most demanding phases in the life of sheep and should have special attention paid to them (Varanis et al., 2021; Antunović et al., 2021, 2022; Singh et al., 2022). The influence of late pregnancy and lactation in sheep on the blood metabolic profile and complete blood count has been extensively studied; however, only a limited number of blood indicators has been included in research projects (Antunović et al., 2004, 2011; Varanis et al., 2021; Singh et al., 2022; Adili et al., 2024; Makgopa et al., 2024). Therefore, this research included a larger number of blood indicators in determining the blood metabolic profile and complete blood count of sheep during late pregnancy and lactation. Since determining the blood metabolic profile and complete blood count of sheep provides a clearer picture of the quality of breeding, possible nutritional errors, and the welfare and health status of the animal, the above will contribute significantly to improving the quality of the herd and optimizing breeding costs. The hypothesis of the present study is that the late pregnancy and lactation period will affect the blood metabolic profile and complete blood count of sheep. This research aims at obtaining the extensive quality information on the selected sheep, so that obtained values could be compared with those already published, and that recommendations could be given for further usage of the blood metabolic profile and complete blood count in extensive sheep breeding.

## 2 Material and methods

### 2.1 Location of research and selection and measurement of sheep

The research was conducted on 30 adult clinically healthy Lika Pramenka sheep kept at the Kučan Agricultural Farm in Lika, Croatia, from December 2025 to April 2026. The farm has been dealing with extensive sheep breeding for many years. The selected sheep were, on average, 5 years old, in the fourth lactation, with a lambing index of 1.2. Sheep for this research were selected from a herd of some 800 sheep. The feeding of sheep was based on meadow hay at an amount of 1.8 $\mathrm{kg}\;d^{-1}$ and oat at an amount of 800 $g\;d^{-1}$ . The sheep were housed in a barn, with 30 animals in a pen. The criteria for selecting the sheep were health, age uniformity, lactation sequence and body condition. Anthelmintic treatments are carried out regularly on the farm. Selected sheep were healthy and weighed with a livestock scale, and the initial average body weight of the selected sheep was 53.36 kg. Determination of the body condition index of sheep was carried out according to Russel (1991), with scores from 1 to 5, and the average initial body condition score was 3.75.

### 2.2 Blood sampling and determination of blood metabolic profile and complete blood count

Blood was sampled from late-pregnancy sheep (on the 20th day before lambing) and during the lactation period on the 40th, 70th and 100th days. Blood was taken from the same sheep through four samplings conducted at the same time ( n=120 blood samples in total). Blood for complete blood count was sampled into a Venoject^®^ sterile vacuum tube (Sterile Terumo Europe, Leuven, Belgium), which contained ethylenediaminetetraacetic acid (EDTA) as an anticoagulant, while blood samples for biochemical parameters were taken in plain tubes (Vacutube, Vodice, Slovenia). After sampling, samples of blood were cooled down to +4 °C and transported to the laboratory of the Faculty of Agrobiotechnical Sciences Osijek, where complete blood counts were determined the same day, and blood smears were made to determine the differential blood count. Determined complete blood count refers to WBCs (white blood cells); RBCs (red blood cells); PLTs (platelets); content of hemoglobin (HGB) and hematocrit (HCT); and the mean corpuscular volume (MCV), the mean corpuscular hemoglobin (MCH) and the mean corpuscular hemoglobin concentration in the erythrocytes (MCHC). Those indicators were determined using a Sysmex PocH-100iV automated three-differential hematology analyzer (Sysmex Europe GmbH, Hamburg, Germany). The percentage of leukocytes, namely neutrophils, lymphocytes, basophils, eosinophils and monocytes, was determined within the differential blood count by means of a Pappenheim stain. Then the blood samples were centrifuged for 10 min at 1609.92×g in a ROTOFIX 32A centrifuge (Hettich GmbH & Co. KG, Tuttlingen, Germany). The obtained serum was frozen at -80 °C . Biochemical analysis of the sheep blood serum was performed in an automatic Olympus AU 400 (Olympus, Tokyo, Japan). Concentrations of minerals (Ca, P-inorganic and Fe), urea, glucose, total proteins, albumins, cholesterol, HDL-cholesterol (high-density lipoprotein), LDL-cholesterol (low-density lipoprotein), triglycerides and NEFA (non-esterified fatty acids) were determined, along with enzyme activities: ALT (alanine aminotransferase), AST (aspartate aminotransferase), GGT ( γ -glutamyl transferase), ALP (alkaline phosphatase) and CK (creatine kinase). The globulin content (GLOB) was calculated as the difference between the total protein and albumin content. The ratio of albumin / globulin was calculated as the difference between contents of albumins and globulins. The content of VLDL-cholesterol (very-low-density lipoprotein) in the blood of sheep was calculated using the equation proposed by Friedewald et al. (1972): $\text{VLDL}\;(\mathrm{mmol}\;L^{-1})=\text{Triglycerides}/5$ .

### 2.3 Statistical analysis

Research data were processed using the statistical software SAS^®^ 9.4 (SAS Institute Inc., Cary, NC, USA). The distribution of obtained data was verified by the Shapiro–Wilk test (PROC UNIVARIATE). The results were obtained using the MEANS procedure and were expressed as the arithmetic mean (mean), the standard error of the mean (SEM), while a significant effect of time measurement was obtained by the PROC GLM (general linear model). Significant differences between late-pregnancy and different lactation periods were compared using the Tukey test, and differences between the groups were declared to be significant at P<0.05 and were marked with small letters (a, b, c, d). Pearson's correlation coefficients were used to determine the association between certain variables. Values of the correlation coefficient ( r ) and its description were as follows: 0.00–0.19, very weak; 0.20–0.39, weak; 0.40–0.59, moderate; 0.60–0.79, strong; and 0.80–1.0, very strong. This is as described in Soeharsono et al. (2020).

## 3 Results

### 3.1 Complete blood count of sheep during late pregnancy and lactation

When analyzing the influence of late pregnancy and lactation on complete blood count (Table 1), it was confirmed that these had a significant influence on most of the complete blood count. When compared to lactating sheep, late-pregnancy sheep had significantly higher RBCs and HGB and HCT contents, as well as a higher portion of lymphocytes, but lower PLT counts and portions of neutrophils. Likewise, during lactation, a significant decrease in RBCs, HGB and HCT and in the portion of lymphocytes was found in the second measurement. The PLT count and MCH content were increased, along with the portion of neutrophils, in the blood of the studied sheep.

**Table 1:** Influence of late pregnancy and lactation on complete blood count ( n=30 ).

Indicator	Late pregnancy	Lactation (time of measurement)			SEM	P values
		First (40th days)	Second (70th days)	Third (100th days)		
WBC ( $\times 10^{9}$ L)	8.77	9.79	8.05	10.27	0.395	0.214
RBC ( $\times 10^{12}$ L)	8.03^a^	7.01^a^	5.56^b^	7.57^a^	0.214	0.0002
HGB ( $g\;L^{-1}$ )	95.63^a^	82.57^ab^	67.70^b^	87.90^a^	2.445	0.0005
HCT (%)	0.34^a^	0.30^a^	0.24^b^	0.31^a^	0.009	0.0007
MCV (pg)	42.39	44.01	42.53	40.63	0.453	0.055
MCH (fL)	11.97^ab^	11.92^ab^	12.20^a^	11.64^b^	0.078	0.028
MCHC ( $g\;L^{-1}$ )	284.57	272.35	280.70	288.60	2.845	0.253
PLT ( $\times 10^{9}$ L)	216.23^a^	394.77^b^	430.10^c^	261.57^a^	15.187	<0.0001
Leukocyte distribution, %						
Eosinophils	5.73	5.97	6.60	6.83	0.356	0.734
Neutrophils	28.33^a^	38.86^b^	48.83^c^	39.03^b^	1.046	<0.0001
Lymphocytes	63.83^a^	52.88^b^	42.63^c^	52.17^d^	1.109	<0.0001
Basophils	1.34	1.05	1.07	0.94	0.122	0.635
Monocytes	0.77	1.24	0.87	1.03	0.108	0.641

Mean – average value; SEM – standard error of mean; WBC – white blood cell; RBC – red blood cell; HGB – hemoglobin; HCT – hematocrit; MCV – mean corpuscular volume; MCH – mean corpuscular hemoglobin; MCHC – mean corpuscular hemoglobin concentration; PLT – platelet. ^a, b, c, d^ Row means with different superscripts differ significantly at P<0.05 .

### 3.2 Blood metabolic profile of sheep during late pregnancy and lactation

Late pregnancy and lactation of sheep influenced most of the biochemical indicators (Table 2) in the sheep's blood. In the blood of late-pregnancy sheep, there was a significantly higher concentration of glucose and triglycerides and a higher A / G ratio and significantly lower concentrations of urea, TP, ALB, GLOB, Ca and Fe, as well as lower activity of GGT, CK and AST, as compared to lactating sheep. Also, during lactation, sheep had significantly lower glucose and A / G ratios but increased concentrations of urea, TP, ALB, GLOB, HDL-cholesterol, Ca, P-inorganic and CK activity.

**Table 2:** Influence of late pregnancy and lactation on biochemical parameters of sheep ( n=30 ).

Indicator, $\mathrm{mmol}\;L^{-1}$	Late pregnancy	Lactation (time of measurement)			SEM	P values
		First (40th days)	Second (70th days)	Third (100th days)		
Glucose	2.64^a^	2.46^a^	1.93^b^	1.25^c^	0.062	<0.0001
Urea	2.81^a^	3.16^a^	5.88^b^	6.39^b^	0.177	<0.0001
Total protein, $g\;L^{-1}$	60.27^a^	57.27^a^	60.35^a^	72.59^b^	0.871	<0.0001
Albumin, $g\;L^{-1}$	24.40^a^	22.41^a^	22.26^a^	26.73^b^	0.325	<0.0001
Globulin, $g\;L^{-1}$	35.86^a^	34.86^a^	38.09^a^	45.59^b^	0.618	<0.0001
A / G	0.69^a^	0.68^ab^	0.59^b^	0.60^b^	0.01	0.0021
Cholesterol	1.65^a^	1.68^a^	1.33^b^	1.71^a^	0.088	<0.0001
HDL-cholesterol	1.01^a^	1.02^a^	0.89^b^	1.12^c^	0.017	<0.0001
LDL-cholesterol	0.54^a^	0.59^a^	0.37^b^	0.51^b^	0.013	<0.0001
VLDL-cholesterol	0.04	0.04	0.04	0.04	0.001	0.0570
Triglycerides	0.23^a^	0.21^a^	0.20^a^	0.18^b^	0.006	0.027
NEFA	0.51^a^	0.66^ab^	0.40^b^	0.51^b^	0.032	0.007
Ca	2.10^a^	2.10^a^	2.14^a^	2.45^b^	0.034	0.0002
P-inorganic	1.49^a^	2.29^b^	1.25^a^	1.98^c^	0.050	<0.0001
Fe, $µ\mathrm{mol}\;L^{-1}$	21.36^ab^	23.87^a^	19.65^b^	18.91^b^	0.588	0.014
GGT, $U\;L^{-1}$	46.72^a^	57.43^b^	54.72^c^	57.90^b^	1.080	<0.0001
CK, $U\;L^{-1}$	121.90^a^	134.60^ab^	191.93^b^	191.78^b^	8.379	0.003
ALP, $U\;L^{-1}$	83.47	84.80	110.11	94.80	5.157	0.186
AST, $U\;L^{-1}$	94.27^a^	113.99^b^	114.91^b^	126.76^b^	2.303	<0.0001
ALT, $U\;L^{-1}$	16.88	17.20	17.87	19.87	0.384	0.054

Mean – average value; SEM – standard error of mean; HDL-cholesterol – high-density lipoprotein; LDL-cholesterol – low-density lipoprotein; VLDL-cholesterol – very-low-density lipoprotein; NEFA – non-esterified fatty acids, ALT – alanine aminotransferase, AST – aspartate aminotransferase, GGT – γ -glutamyl transferase, ALP – alkaline phosphatase; and CK – creatine kinase. ^a, b, c^ Row means with different superscripts differ significantly at P<0.05 .

### 3.3 Correlation between complete blood count and blood metabolic profile of sheep during late pregnancy and lactation

Table 3 refers to heatmaps of correlations between the complete blood counts of the sheep blood. Numerous significant positive and negative correlations were established between the investigated complete blood count in the sheep blood. A very strong positive correlation between RBC : HGB ( r=0.98 ), RBC : HCT ( r=0.97 ) and HGB : HCT ( r=0.95 ) should be highlighted, as well as a very strong negative correlation with NEUT : LIM ( r=-0.94 ). Furthermore, there was strong positive correlation between WBC : RBC ( r=0.63 ), WBC : HGB ( r=0.64 ) and WBC : HCT ( r=0.64 ) established in the sheep's blood.

**Table 3:**
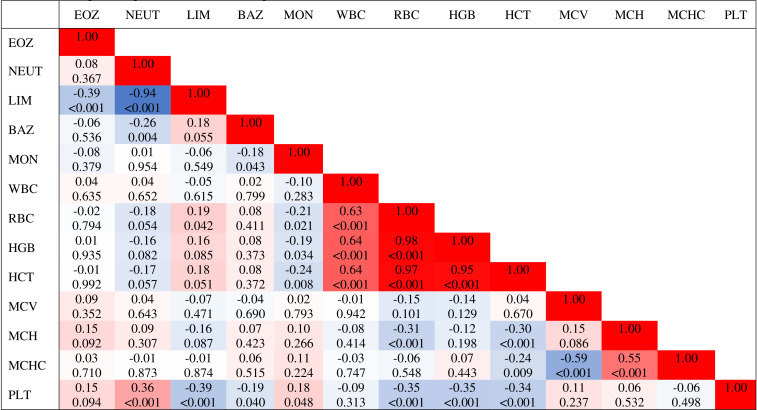
Heatmap of complete blood count of sheep.

WBC – white blood cells; RBC – red blood cells; HGB – hemoglobin; HCT – hematocrit; MCV – mean corpuscular volume; MCH – mean corpuscular hemoglobin; MCHC – mean corpuscular hemoglobin concentration; PLT – platelet; EOZ – eosinophils; NEUT – neutrophils; LIM – lymphocytes; BAZ – basophiles; MON – monocytes.

Table 4 shows heatmaps of correlations between biochemical indicators in the sheep's blood. There were many significantly positive but also some negative correlations determined between biochemical indicators in the blood of sheep during late pregnancy and lactation. Very strong positive correlation was determined between CHOL : HDL ( r=0.87 ), CHOL : LDL ( r=0.77 ); TPROT : ALB ( r=0.70 ) and TPROT : GLOB ( r=0.91 ). Strong negative correlation was found for GLOB : AG ( r=-0.60 ).

**Table 4:**
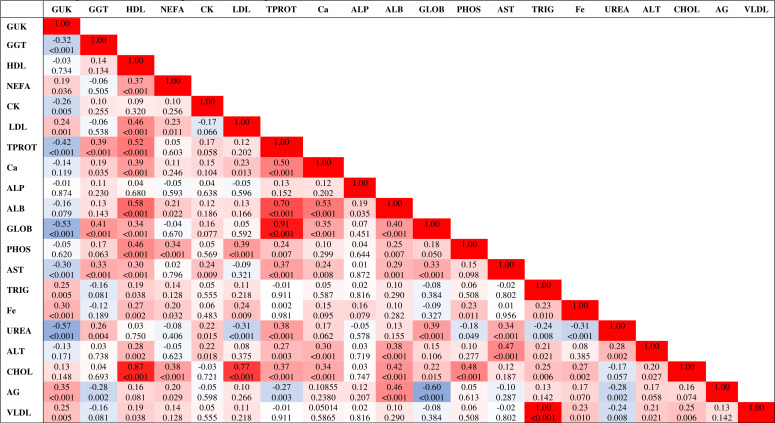
Heatmap of blood biochemical parameters of sheep.

GUK – glucose; CHOL – cholesterol; HDL-cholesterol – high-density lipoprotein; LDL-cholesterol – low-density lipoprotein; VLDL-cholesterol – very-low-density lipoprotein; NEFA – non-esterified fatty acids; ALT – alanine aminotransferase; AST – aspartate aminotransferase; GGT – γ -glutamyl transferase; ALP – alkaline phosphatase; CK – creatine kinase; TPROT – total protein; ALB – albumins, GLOB – globulins; AG – ratio albumin/globulins; TRIG – triglycerides; PHOS – phosphor-inorganic.

## 4 Discussion

### 4.1 Complete blood count of sheep during late pregnancy and lactation

The determination of indicators of the blood metabolic profile and complete blood count is gaining importance with regard to ensuring the best possible herd supervision and feeding management and to provide for the highest possible animal productivity and diagnostics of diseases (Hernandez et al., 2020; Saravanan et al., 2020). The influence of late pregnancy and lactation on the complete blood count of sheep, through determination of the complete blood count, was confirmed in this research (Table 1). Compared to lactating sheep, late-pregnancy sheep had significantly higher RBCs, as well as higher contents of HGB and HCT. The portion of lymphocytes was also higher; however, there was a lower PLT count and a lower portion of neutrophils. The stated changes could be associated with significant changes that occur in late-pregnancy sheep due to the fast development of the foetus and the adaptation of the organism to direct the metabolism of nutrients for foetus development. The lowest RBCs, the content of HGB and HCT, in the blood of lactating sheep during the second measurements could indicate anaemia (Roland et al., 2014).

In research performed in Brazil, Santarosa et al. (2022) also found similar changes in RBCs, HGB, MCV, MCHC and lymphocytes in Dorper ewes on the 140th day of pregnancy. Makgopa et al. (2024) emphasized the need to determine the influence of the pregnant or non-pregnant status on the complete blood count in the sheep's blood. Namely, RBCs and the content of HGB and HCT indicate the body's ability to transport oxygen (Patel, 2008). Salem (2017) reported similar significant reductions in RBCs and HGB and HCT content in lactating ewes. Anwar et al. (2012) argued that the content of HCT in the first months of lactation suggested that the elevated RBC devastation in mammary cells was responsible for reduced HCT, along with the mobilization of water to the mammary gland. In the present study, we found a decrease in the erythrocyte count and HGB content in the blood of ewes during lactation, especially during early lactation, in comparison with reference values (Baumgartner and Wittek, 2017; for RBC: 9– $15\times 10^{12}$ L; for HGB: 90–150 $g\;L^{-1}$ ). These changes may be a consequence of hemodilution due to physiological adaptations in early lactation, primarily caused by increased needs for milk synthesis (El-Sherif and Assad, 2001). It is known that ruminants in the early stage of lactation are often faced with an energy deficit because of fat mobilization. This occurs when milk production is at its peak, which poses additional stress on the animal because high-intensity metabolism causes oxidative stress and exposes animals to infections. Blanco et al. (2009) pointed out that stress and significantly lower intake of protein through feed could also lead to anemia in dairy cows. Yang et al. (2024) stated that cows in early lactation could have a compromised immunity system, which made them susceptible to bacterial infection and anemia. Those authors also pointed out that good feeding management could prevent such an influence during late pregnancy and lactation. As emphasized by Cole et al. (1997), anaemia in ruminants is a common abnormality that is detected by blood count, and it is diagnosed when HCT is less than 24 %, $\text{RBC}<5\times 10^{6}$ ${\mathrm{mL}}^{-1}$ or the content of HGB<80 $g\;L^{-1}$ , which was confirmed in the second measurement of lactation period in this research, while, in other measurements, there were no such indications. Under similar farming conditions, Shek Vugrovečki et al. (2017) determined similar average contents of HGB (89.6 $g\;L^{-1}$ ) and HCT (0.27 $L\;L^{-1}$ ) and a higher content of MCHC in the blood of anoestrous Lika Pramenka sheep; in our research, these were below reference values. In this research, lower average RBCs and HGB content were also determined. In this research, during the second blood measurement, lactating sheep had pronounced slight anemia ( $\text{RBC}=5.56\times 10^{12}$ L; HGB=67.70 $g\;L^{-1}$ ; HCT=0.24% ). When comparing the measured mean values with the reference values for sheep (Baumgartner and Wittek, 2017), this research resulted in a higher average value of MCV, while MCHC was below the reference value. Such changes indicated macrocytosis and hypochromia of sheep (Jain, 1993). During the puerperium period, neutrophils were increased. Sheep in the late-pregnancy, postpartum and puerperium stages are under elevated stress if compared to other reproductive stages (Bezerra et al., 2017). In this research, the lymphocyte portion in sheep blood was lowered as the lactation progressed, which was in accordance with the findings of Antunović et al. (2022) for lactating Lacaune sheep. However, it should be emphasized that, in this research, the indicators of the differential blood count did not deviate from the reference values for sheep (Latimer et al., 2003; for neutrophils: 10 %–50 %; for lymphocytes: 50 %–75 %). Namely, Yaqub et al. (2013) pointed out that the neutrophil / lymphocyte ratio was used to assess stress in animals. In the study of Bezerra et al. (2017), lymphocytes in the blood of sheep showed a tendency to decrease moderately with the progress of pregnancy and the proximity of delivery.

### 4.2 Blood metabolic profile of sheep during late pregnancy and lactation

Analysis of biochemical indicators in the blood showed a significant influence of late pregnancy and lactation in sheep (Table 2). Compared to sheep in lactation, late-pregnancy sheep had significantly higher concentrations of glucose and triglycerides in the blood. The mentioned changes in glucose concentrations, which were below the reference values (Kaneko et al., 2008: 2.78–4.44 $\mathrm{mmol}\;L^{-1}$ ), especially in lactating sheep, as well as changes in NEFA concentrations, indicate the energy deficit.

Compared to measurements during lactation, significantly higher blood glucose concentrations in late-pregnancy sheep as determined in this research were in line with the results obtained by Husted et al. (2008) for sheep in the last 3 weeks of pregnancy; these also decreased by the eighth week of lactation. Reynolds et al. (2003) also reported that the glucose concentration in the blood of late-pregnancy cows was significantly higher than that in lactating cows, which was explained by increased hepatic gluconeogenesis. Drackley et al. (2001) stated that the demand for glucose increased significantly after calving, and cows fulfilled that demand through gluconeogenesis using propionates, amino acids and glycerol. Kour et al. (2025) linked such changes in blood glucose in lactating cows to decreased energy intake.

The highest concentrations of NEFA in the present research were found in the blood of sheep in early lactation. This is also associated with a decrease in glucose concentrations in the blood of lactating sheep as the metabolism adjusts itself to fulfill the energy demands of the growing fetus and the mammary gland for lactogenesis (Samira et al., 2016). Singh et al. (2022) also reported similar changes in NEFA concentrations, as well as opposing changes in glucose concentrations in the blood of late-pregnancy sheep and lactating sheep. As stated by Castagnino et al. (2015), in this state, the body uses glycogen stores in the liver to produce glucose and switches to mobilizing large amounts of body fat reserves to compensate for the resulting deficit, which results in the production of ketone bodies. However, when ketone concentrations increase in the body, glucose concentrations fall (Hernandez et al., 2020). Energy imbalances occur often in late-pregnancy sheep and sheep in early lactation, and it is induced because of fetal growth and milk synthesis. In this research, NEFA levels were within physiological ranges, set by Reintke et al. (2021) to 0.1–0.5 $\mathrm{mmol}\;L^{-1}$ , except on the 30th day of lactation.

This research confirmed a significant decrease in the concentration of triglycerides in the blood of lactating sheep, which could be associated with increased lipogenesis during lactation and with energy imbalance due to the fact that triglycerides are the main element in milk synthesis. Mazur et al. (2009) published similar conclusions.

As pointed out by Araujo et al. (2015), proteins in the blood can indicate certain metabolic functions of the intestine and the state of health itself. Furthermore, it is known that ALB concentrations in the blood are an indicator of the long-term protein status in animals, while TP concentrations in the blood are a good indicator of protein reserves in the body. Significant reductions in urea concentrations, TP, ALB and GLOB in the blood of late-pregnancy sheep and sheep in early lactation confirm all of the above-stated results. Lowered concentrations of TP, ALB and GLOB in the blood of late-pregnancy sheep and sheep in early lactation, as confirmed in this research, are in accordance with results published by El-Sherif and Assad (2001). As reported by Schmitt et al. (2018), protein is the main source of uterine tissues in late-pregnancy sheep, and the foetus synthesizes all of its protein from amino acids derived from the sheep. In the first measurement during lactation, urea concentrations in the blood of late-pregnancy and lactating sheep were at the lower limit of the reference values (Kaneko et al., 2008: 2.86–7.14 $\mathrm{mmol}\;L^{-1}$ ), which indicated a lack of protein in the feed. Such a conclusion is also supported by the determined lower concentrations of TP and ALB in the blood of late-pregnancy and lactating sheep in the first two measurements during lactation. Those concentrations were at the lower limit of or below the reference values (Kaneko et al., 2008; for TP: 60–79 $g\;L^{-1}$ ; for ALB: 24–30 $g\;L^{-1}$ ), while the A / G ratio was the highest in the late-pregnancy sheep and in lactating sheep in the first measurement. In their study on lactating cows, Bobbo et al. (2017) concluded that, in intensive production systems, feeding regimes could significantly affect ALB concentrations in the animal blood. In their research on fat-tailed sheep during different physiological stages, Sarmin et al. (2021) found that sheep in lactation had higher concentrations of GLOB than late-pregnancy sheep, which was explained by an increase in α -globulins (Bobbo et al., 2017). In this research, late-pregnancy sheep had higher A / G ratios in their blood than lactating sheep during the second and third measurements. On the contrary, Sarmin et al. (2021) measured higher A / G ratios in the blood of lactating sheep than in pregnant sheep (1.19 : 0.93, respectively). In the first two measurements, determined changes in the protein status in sheep's blood, especially in late-pregnancy and lactating sheep, indicated dysproteinemia (Kaneko et al., 2008).

Lower concentrations of Ca and Fe in the blood of late-pregnancy and lactating sheep indicated their increased consumption during the intensive growth and development of the foetus, yet the lack of Ca and Fe could also occur because of increased milk synthesis in lactating sheep, especially in its first part. Such occurrence was also reported in the research of Castillo et al. (1997) as they argued that mobilization of Ca and P from bone followed a similar mechanism and that Ca got deployed from blood to milk so that P could remain stable or even elevated in the blood if compared to Ca. Liesegang et al. (2007) pointed out that a reduced Ca concentration in blood during late pregnancy and early lactation resulted in the increased Ca requirement for foetal secretion and the increased Ca requirement for milk secretion. Similar changes in P-inorganic concentrations in sheep's blood were also found in the present research. A significant decrease in Fe concentrations in the blood of lactating sheep in the present research indicated the excretion of Fe in milk. Such changes in P-inorganic concentrations and the significant decrease in Fe concentrations in the blood of lactating sheep were also reported by Singh et al. (2022).

Significantly lower activity of enzymes (GGT, CK and AST) in the blood of late-pregnancy sheep and their increase during lactation indicated the overload of the organism and of the liver itself with protein deamination, which was related to the increased needs for milk secretion. Most enzyme activities in sheep during late pregnancy and lactation, except GGT during lactation, were within the reference limits (Kaneko et al., 2008). As obtained in the present study, increased activities of ALT, AST and GGT were determined in the blood of lactating sheep. This is associated with more intense metabolic activities in the liver and the body reacting to a negative energy balance (Stevanović et al., 2015; Hrković-Porobija et al., 2017). The identified changes in the activity of the stated enzymes in the present research could be connected with the reduced dry matter intake and increased fat mobilization around the time of lambing, which led to hepatic lipidosis and affected the normal liver status (Greenfield et al., 2000). The AST activity in the blood of sheep impacts the estimation of the liver status and plays an important role in the metabolism of amino acids (Donia et al., 2014). Such a condition requires more protein and energy due to increased milk secretion in lactating sheep as the liver is intensively burdened (Roubies et al., 2006).

### 4.3 Correlation between complete blood count and blood metabolic profile of sheep during late pregnancy and lactation

This research established numerous significant positive and negative correlations between the investigated complete blood count and biochemical indicators in the blood of sheep during late pregnancy and lactation, which indicated their mutual dependence in relation to the sheep's metabolism (Tables 3 and 4). There were very strong positive correlations established between complete blood count (RBC : HGB ( r=0.98 ), RBC : HCT ( r=0.97 ) and HGB : HCT ( r=0.95 )), as well as between blood biochemical parameters (CHOL : HDL ( r=0.87 ), CHOL : LDL ( r=0.77 ), TPROT : ALB ( r=0.70 ), TPROT : GLOB ( r=0.91 )). The significant positive correlation found between RBC, HGB and HCT is physiologically expected, as are the correlations between indicators of protein (TP, ALB and GLOB) and lipid status (CHOL, HDL and LDL). Similar results regarding the strong positive correlation between RBC : HCT ( r=0.93 ) and RBC : WBC ( r=0.47 ) in sheep's blood were published by Makgopa et al. (2024). Results obtained in this research are in accordance with those published by Fanta et al. (2024), who reported that RBCs were positively correlated with WBCs and HCT in Doyogena sheep in Ethiopia. HCT and RBCs are related through HCT, which measures the percentage of blood made up of RBCs (Etim et al., 2014).

## 5 Conclusion

Late pregnancy and lactation have an important influence on indicators of the sheep's blood metabolic profile and complete blood count. Their influence is especially significant in late-pregnancy and lactating sheep during the first measurement. During these stages, this research confirmed significant changes in complete blood count (RBCs, HGB, HCT, PLT, lymphocytes and neutrophils), as well as in indicators of the energy status (NEFA, glucose and triglycerides), protein status (TP, ALB, urea), concentration of minerals (Ca, P-inorganic) and liver status (changes in most liver enzymes). Moreover, the quality of nutrition (protein and mineral content) plays an important role in the proper definition of the blood metabolic profile and complete blood count of sheep during late pregnancy and lactation.

## Data Availability

The data are available from the corresponding author upon reasonable request.
